# Effect of Temperature on Cystic Fibrosis Lung Disease and Infections: A Replicated Cohort Study

**DOI:** 10.1371/journal.pone.0027784

**Published:** 2011-11-18

**Authors:** Joseph M. Collaco, John McGready, Deanna M. Green, Kathleen M. Naughton, Christopher P. Watson, Timothy Shields, Scott C. Bell, Claire E. Wainwright, for the ACFBAL Study Group, Garry R. Cutting

**Affiliations:** 1 Eudowood Division of Pediatric Respiratory Sciences, Johns Hopkins University School of Medicine, Baltimore, Maryland, United States of America; 2 Department of Biostatistics, Johns Hopkins Bloomberg School of Public Health, Baltimore, Maryland, United States of America; 3 McKusick-Nathans Institute of Genetic Medicine, Johns Hopkins University School of Medicine, Baltimore, Maryland, United States of America; 4 Department of Molecular Microbiology and Immunology, Johns Hopkins Bloomberg School of Public Health, Baltimore, Maryland, United States of America; 5 The Prince Charles Hospital and University of Queensland, Brisbane, Queensland, Australia; 6 Royal Children's Hospital and University of Queensland, Brisbane, Queensland, Australia; Abramson Research Center, United States of America

## Abstract

**Background:**

Progressive lung disease accounts for the majority of morbidity and mortality observed in cystic fibrosis (CF). Beyond secondhand smoke exposure and socio-economic status, the effect of specific environmental factors on CF lung function is largely unknown.

**Methods:**

Multivariate regression was used to assess correlation between specific environmental factors, the presence of pulmonary pathogens, and variation in lung function using subjects enrolled in the U.S. CF Twin and Sibling Study (CFTSS: n = 1378). Significant associations were tested for replication in the U.S. CF Foundation Patient Registry (CFF: n = 16439), the Australian CF Data Registry (ACFDR: n = 1801), and prospectively ascertained subjects from Australia/New Zealand (ACFBAL: n = 167).

**Results:**

In CFTSS subjects, the presence of *Pseudomonas aeruginosa* (OR = 1.06 per °F; *p*<0.001) was associated with warmer annual ambient temperatures. This finding was independently replicated in the CFF (1.02; *p*<0.001), ACFDR (1.05; *p* = 0.002), and ACFBAL (1.09; *p* = 0.003) subjects. Warmer temperatures (−0.34 points per °F; *p* = 0.005) and public insurance (−6.43 points; *p*<0.001) were associated with lower lung function in the CFTSS subjects. These findings were replicated in the CFF subjects (temperature: −0.31; *p*<0.001; insurance: −9.11; *p*<0.001) and similar in the ACFDR subjects (temperature: −0.23; *p* = 0.057). The association between temperature and lung function was minimally influenced by *P. aeruginosa*. Similarly, the association between temperature and *P. aeruginosa* was largely independent of lung function.

**Conclusions:**

Ambient temperature is associated with prevalence of *P. aeruginosa* and lung function in four independent samples of CF patients from two continents.

## Introduction

Cystic fibrosis (CF) is an autosomal recessive disorder caused by mutations in the *CFTR* gene. Progressive obstructive lung disease and recurrent respiratory infections account for the majority of morbidity and mortality associated with CF. Significant variation in CF lung disease exists, even among individuals with identical mutations [1]–[3], and approximately half of this variation is attributable to environmental and/or stochastic (random) factors [4], [5]. To improve outcomes for this life-limiting disorder, it is important to identify specific environmental factors that ameliorate or exacerbate disease. Prior CF studies have demonstrated associations between lung function and local environmental factors, including secondhand smoke exposure [6]–[10], air pollution [11], household income [12]–[14], maternal education [13], and insurance status [13], [15]–[17]. However, with the exception of air pollution [11], there has been limited work examining environmental factors operating on a geographic scale, such as climate.

Using data from the U.S. CF Twin and Sibling Study, we sought to determine (i) whether selected geographic factors (factors mapped using geospatial techniques) were associated with the presence of significant respiratory pathogens in CF, (ii) whether geographic factors were associated with variation in lung function, and (iii) the relative impact of geographic factors compared to other environmental factors (demographic or household) in multivariate analyses. We attempted to replicate key findings in subjects from two national CF patient registries (United States and Australia). The role of infection was also examined in a prospective sample of CF patients from Australia and New Zealand.

## Methods

### Ethics Statement

Written informed consent was obtained from CFTSS subjects. This study, including the CFF, ACFDR, and ACFBAL data downloads, were specifically approved by the Johns Hopkins University Institutional Review Board (NA_00035659, NA_00019677). **Participants: U.S. CF Twin and Sibling Study (CFTSS):** Subjects were recruited from CF centers based on having a twin or sibling also with CF (n = 1658 in 817 families) [18]. Data were collected between 10/27/00 and 9/25/09 with data supplementation from the U.S. CF Foundation Patient Registry through 12/31/08. **U.S. CF Foundation Patient Registry (CFF):** Anonymized data from the calendar year 2007 was provided (n = 24,799). **Australian Cystic Fibrosis Data Registry (ACFDR):** Anonymized data from 1998–2008 was provided (n = 3789). **Australian Cystic Fibrosis Bronchoalveolar Lavage Trial (ACFBAL):** Anonymized data was provided for 168 infants diagnosed with CF in Australia and New Zealand between 6/10/99 and 1/18/05 who were prospectively followed for microbial acquisition until 5 years of age. Subjects were excluded if lung function (CFTSS, CFF, ACFDR), respiratory culture, or residential postal/zip code data were not available, or if actively smoking (CFTSS, CFF)(Figures S1 and S2). CFTSS subjects enrolled in the CFF Patient Registry (n = 1435) were excluded from the CFF sample.

### Household Variables

Secondhand smoke exposure was defined as any reported home exposure (Table 1). Maternal education was characterized as 1 = less than a high school education; 2 = high school graduate; 3 = some college education; 4 = college graduate. Household income was estimated using zip code and 2000 U.S. Census data. Insurance status was determined from the most recent year of data. Household density was the number of persons residing within the household. Household data were not available for Australian subjects.

**Table 1:** Study Sample Demographics.

	Variable (Mean ± SD) [Range]	CFTSS (n = 1378)	CFF (n = 16439)	ACFDR (n = 1801)	ACFBAL (n = 167)
Demographics	Sex (% Female)	48.7	47.9	46.9	47.3
	CFTR Genotype (% *F508del* homozygote)^1^	58.6 (n = 1371)	50.0 (n = 14733)	51.2 (n = 1646)	66.5
	Race/Ethnicity (% Non-Hispanic White only)^2^	90.9	88.8 (n = 16406)	-	95.2
	Age at Diagnosis (yrs)^3^	2.3±5.4 [0 – 52.0]	3.8±8.2 [0 – 73.7]	2.0±6.5 [0 – 67.3] (n = 1578)	0.1±0.1 [0 – 0.4]
	Age at time of lung function test (yrs)^4^	17.4±8.9 [6.0 – 63.2]	20.5±11.4 [6.0 – 74.0]	20.2±10.6 [6.0 – 76.1]	-
	Age at time of last respiratory culture (yrs)^5^	17.4±9.0 [5.3 – 63.9]	20.8±11.4 [5.7 – 74.2]	19.3±10.4 [0 – 76.0]	4.9±0.8 [0.6 – 6.3]
Household Factors	Secondhand Smoke (% Exposed)^6^	33.8 (n = 1313)	29.4 (n = 8477)	-	-
	Maternal Education (Scale: 1–4)^7^	3.0±.0 (n = 1296)	3.0±1.0 (n = 6874)	-	-
	Income (log $)^7^	4.67±0.16[4.15 – 5.19]	4.65±0.15[3.87 – 5.26](n = 15977)	-	-
	Insurance Status (%)^7^No InsuranceAny state MAOther	2.839.457.9(n = 1357)	1.239.759.1(n = 16258)	-	-
	Household Density (persons/household)^7^	4.2±1.7[1]–[12](n = 1275)	3.4±1.4[1]–[12](n = 11847)	-	-
Geographic Factors(by residential zip code)	PM_2.5_ level (µg/m^3^)	11.9±2.5[4.8 – 22.4](n = 677)	11.9±2.6[3.4 – 22.4](n = 7698)	-	-
	Elevation (log m)^7^	2.13±0.63[0 – 3.30](n = 1372)	2.08±0.67[0 – 3.51](n = 15532)	-	-
	Relative Humidity (%)^7^	67.6±5.6[43.5 – 85](n = 1372)	66.7±6.1[34 – 83](n = 15532)	-	-
	Temperature (°F)^7,8^	53.6±7.1[37.8 – 75.0](n = 1372)	55.3±7.8[34.3 – 77.5](n = 15532)	63.3±4.8[46.1 – 80.8](n = 1791)	62.9±5.9[49.8 – 81.2](n = 166)
	Distance from Care (log Km)^7^	1.68±0.60[−1 – 3.61](n = 1377)	1.63±0.60[−1 – 3.77](n = 16121)	-	-
	Population Density(log persons/km^2^)	2.31±0.78[−0.45 – 4.53](n = 1364)	2.35±0.80[−1.15 – 4.70](n = 15973)^4^	-	-
Outcomes	*P. aeruginosa* (% Positive)^9^	87.9	60.5	80.3	60.5
	First *P. aeruginosa* Positive Culture (yrs)^10^	6.7±6.3[0.2 – 49.9](n = 919)	-	-	2.3±1.4[0.1 – 5.3](n = 101)
	Mucoid *P. aeruginosa* (% Positive)^7^	61.3	42.8	-	-
	First Mucoid *P. aeruginosa* Positive Culture (yrs)	11.3±7.9[0.3 – 53.8](n = 797)	-	-	-
	*B. cepacia* complex (% Positive)^11^	7.7	3.4	6.8	-
	First *B. cepacia* complex Positive Culture (yrs)	13.8±8.4[1.0 – 43.9](n = )	-	-	-
	Lung Function (CF-specific FEV_1_ Percentile)^12^	69.3±26.3	65.3±26.4	62.8±24.6	-

1 The ACFBAL sample has a higher frequency of *F508del* homozygotes than other samples (ANOVA *p*<0.001). ^2^The CFF sample has a lower proportion of Non-Hispanic Whites than other samples (ANOVA *p* = 0.002). ^3^CFF and ACFBAL samples diagnosed at older and younger ages, respectively, than other samples (ANOVA *p*<0.001). ^4^The CFTSS sample has a younger age of pulmonary function testing than other samples (ANOVA *p*<0.001). ^5^All samples differ from each other by age of last respiratory culture (ANOVA *p*<0.001). ^6^Definition of secondhand smoke for the CFTSS sample (ever having been exposed) differs from the CFF sample definition (exposure within the past year (2007)). ^7^CFTSS and CFF samples are statistically different (*T* test or chi square *p*<0.01). ^8^Mean temperatures of ACFDR and ACFBAL samples are not statistically different (*T* test *p* = 0.34). ^9^The ACFDR sample has a higher prevalence of *P. aeruginosa* than the CFF and ACFBAL samples, and the CFTSS sample has a higher prevalence than all other samples (ANOVA *p*<0.001). ^10^The ACFBAL sample has an earlier age of acquisition than the CFTSS sample (ANOVA *p*<0.001). ^11^The CFF sample has a lower prevalence of *B. cepacia* complex than other samples (ANOVA *p*<0.001). ^12^All samples differ by mean lung function (ANOVA *p*<0.001).

### Geographic Variables

U.S. subjects were mapped to the center of their most recent known residential zip code (finest resolution available), to derive measures for humidity, temperature, air pollution (Fine particulate matter: PM_2.5_), elevation, and distance to the CF care center of record using ArcGIS 9.3 (ESRI; Redlands, CA) and ERDAS Imagine 9.3 (ERDAS; Atlanta, GA). Source data were selected on the basis the time period available that matched the clinical data and included 1961–1990 mean monthly and annual relative humidity and temperatures (The Climate Source; Corvallis, OR; 2 km resolution), 2006 PM_2.5_ measures (U.S. Environmental Protection Agency), elevations (U.S. Geological Survey), and population densities (2000 U.S. Census). PM_2.5_ measures were derived for only subjects living within 30 miles of a pollution monitor based on previous work by Goss *et al*. [11]. Australian subjects were mapped to the center of their residential postal code to derive measures for mean annual temperature using System for Automated Geoscientific Analyses (SAGA User Group Association; Hamburg, Germany), GDAL libraries and Python bindings (Open Source Geospatial Foundation; Vancouver, BC), and Shapely v1.2 (Python Software Foundation; Wolfeboro Falls, NH). Source data included 1961–1990 and 1971–2000 mean annual temperatures (Australian Bureau of Meteorology and the [New Zealand] National Climate Database, respectively).

### Outcome Variables

Raw FEV_1_ (liters) measurements were converted into CF-specific percentiles [19], excluding measurements obtained before 6 years of age and after lung transplantation. Lung function was defined as the best percentile in the most recent year of data. The higher lung function measured in the CFTSS subjects vs. other subjects likely reflects the younger age of CFTSS subjects (Table 1; Table S1). The lower lung function observed in the ACFDR subjects may represent the limitation of applying of a U.S.-based phenotype to an Australian population. Using all available respiratory culture data, subjects were considered to be positive for an organism (*Pseudomonas aeruginosa,* mucoid *P. aeruginosa,* or *Burkholderia cepacia* complex) if they had any cultures positive for that organism. The higher prevalence of infectious organisms in the CFTSS and ACFDR subjects vs. the CFF subjects reflects the multi-year culture data compared to the single year data, respectively (Table 1). Age of acquisition for an organism was defined as the date of the first positive culture following at least one prior negative culture (CFTSS, ACFBAL) [20]. Means of ascertainment likely account for age of acquisition differences between the retrospective CFTSS and prospective ACFBAL samples.

### Data Analysis

Regressions clustered by family (CFTSS) or by CF care center (CFF), ANOVA, chi square, and student's t-tests were performed using Stata 10 (StataCorp LP; College Station, TX). Center-level data were not available for Australian subjects. CFTSS subjects served as the primary population being the best characterized of the study populations. The CFTSS preliminary multivariate (logistic for presence of pathogens; linear for lung function) regression models included all predictor variables significant in univariate modeling. The final CFTSS models were generated by dropping non-significant (*p*≥0.05) predictors from the preliminary models in a stepwise manner. The final CFTSS models were used in replication regressions for the other samples. For U.S. and Australian subjects, temperature quartiles were derived from all CFTSS subjects (n = 1557) and ACFDR subjects (n = 3635) with temperature data, respectively. Kaplan-Meier plots were constructed for the age of acquisition for *P. aeruginosa* by temperature quartiles for the CFTSS and ACFBAL subjects. The effect of mediation by infection on lung function was estimated by dividing the difference of regression coefficients of temperature adjusted and unadjusted for the mediator by coefficient of temperature unadjusted for the mediator [21], [22]. The mediation effect by lung function on infection was estimated similarly using the logarithms of the temperature odds ratios.

## Results

### Mean Annual Temperature is Associated with Prevalence of and Age at *P. Aeruginosa* Infection

Given the importance of bacterial infection in CF lung function decline [23], [24], we tested whether selected environmental factors were associated with specific organisms using multivariate logistic regression. Among CFTSS subjects, ambient temperature (Table 2: OR = 1.06 per °F; *p*<0.001) was associated with the prevalence of *P. aeruginosa*. *CFTR* genotype (based on the number of the most common mutation (*F508del*) present, OR = 2.05 per *F508del* mutation; *p*<0.001), age at the last respiratory culture (OR = 1.19 per year; *p*<0.001), and age at diagnosis (OR = 0.84 per year; *p*<0.001) were also associated with *P. aeruginosa* prevalence, as previously reported (Complete regression results in Table S2) [3], [20], [25]. In contrast to previous studies [26], [27], none of the household factors tested were associated with the presence of *P. aeruginosa.* The observed association was not accounted for by increased culturing frequency in warmer regions, as a higher frequency of cultures was associated with colder temperatures (Regression coefficient *p*<0.001; n = 1370). Also, the association was not accounted for by a temperature-humidity interaction (Interaction term *p* = 0.45).

**Table 2:** Annual Ambient Temperature is a Predictor of *P. aeruginosa* Infection.^1^

Study	CFTSS	CFF	ACFDR	ACFBAL
Study design (Country)	Retrospective(U.S.)	Retrospective(U.S.)	Retrospective (Australia)	Prospective (Australia)
**N**	1366	13956	1474	166
**Adjusted Odds Ratio for Temperature (per °F) [95%CI]**	1.06[1.03, 1.09]	1.02[1.01, 1.02]	1.05[1.02, 1.08]	1.09[1.03, 1.16]
**Odds Ratio ** ***p*** ** Value**	<0.001	<0.001	0.002	0.003

1 Complete results from multivariable regression analyses, including adjustments, can be found in Table S2. *CFTR* genotype, age at the time of the last respiratory culture, and age at diagnosis were all significant predictors of *P. aeruginosa* infection for the CFTSS, CFF, and ACFDR samples, but not the ACFBAL sample.

The association of warmer temperatures and an increased *P. aeruginosa* prevalence was also seen in the CFF subjects (OR = 1.02; *p*<0.001). As both the CFTSS and CFF subjects are U.S.-based, this association could be subject to regional biases in socio-economic status, culture, or clinical care patterns. Australian subjects are ideal for replication of geographic modifiers of health outcomes in CF as, like the U.S., the continent has a European immigration-derived CF population and a wide range of climatic conditions, including temperature. The association of warmer temperatures and the prevalence of *P. aeruginosa* was replicated in both the ACFDR (OR: 1.05; *p* = 0.002) and ACFBAL subjects (OR: 1.09; *p* = 0.003). Only older age was associated with the presence of mucoid *P. aeruginosa* (Table S3: CFTSS, CFF) or *B. cepacia* complex (Table S4: CFTSS, CFF, ACFDR), which corresponds to age-specific rates of prevalence reported in CF [24].

To examine whether the relationship between temperature and *P. aeruginosa* was non-linear (as regression analysis assumes linearity), we examined the prevalence of *P. aeruginosa* by temperature quartiles (Table S5). In all four samples of CF subjects, the prevalence of *P. aeruginosa* was higher in warmer temperature quartiles (*p* values<0.001–0.005) paralleling the results of the regression analysis. In addition, age at acquisition of *P. aeruginosa* in the retrospective CFTSS and prospective ACFBAL samples were 15 and 9 months earlier, respectively, in the warmest temperature quartile compared to the coldest (CFTSS *p* = 0.04; Figure S3: log rank test *p* = 0.006; ACFBAL *p* = 0.04; log rank test *p* = 0.001). There were no replicated associations between temperature quartile and prevalence or age of acquisition of mucoid *P. aeruginosa* or *B. cepacia* complex.

### Mean Annual Temperature is Associated with Lung Function

We examined the role of environmental factors on lung function among CFTSS subjects and found that warmer temperatures (−0.34 per °F; *p* = 0.005) were associated with lower lung function (Table 3; complete regression results in Table S1). Although the lung function measure used does account for age [19], older age was associated with lower lung function (−0.40 per year; *p*<0.001), likely due to cohort or survival effect. Public insurance (vs. private insurance) was also associated with lower lung function (−6.43; *p*<0.001), consistent with prior studies [13], [15], [16].

**Table 3:** Annual Ambient Temperature and Insurance Status are Predictors of Lung Function (CF-specific FEV_1_).^1^

Study	CFTSS	CFF		ACFDR
Subjects	All Available	All Available	White, *F508del* homozygotes	All Available
**n**	1313	15174	6367	1791
**Adjusted Co-efficient for Temperature (per °F) [95%CI]**	−0.34[−0.57, −0.10]	−0.31[−0.41, −0.21]	−0.35[−0.46, −0.23]	−0.23[−0.47, 0.01]
**Temperature Co-efficient ** ***p*** ** Value**	0.005	<0.001	<0.001	0.057
**Adjusted Co-efficient for Insurance Status (0 = Private, 1 = Public) [95%CI]**	−6.43[−9.68, −3.19]	−9.11[−10.44, −7.79]	−8.26[−9.90, −6.62]	Not Applicable
**Insurance Co-efficient ** ***p*** ** Value**	<0.001	<0.001	<0.001	-

1 Complete results from multivariable regression analyses, including adjustments, can be found in Table S1. Age at the time of pulmonary function testing was a significant predictor of lung function for the CFTSS and CFF samples, but not the ACFDR sample.

Temperature also was associated with lung function in the CFF subjects (−0.31; *p*<0.001), with a similar magnitude to the CFTSS (−0.34). This corresponds to ∼3 percentile point drop in FEV_1_ for each 10°F increase in mean annual temperature. In addition, warmer temperatures tended to be associated with lower lung function in the ACFDR subjects (−0.23; *p* = 0.057). Mean annual temperatures in Australia are higher than in the U.S., which may influence the magnitude of the coefficient and its significance. The lower lung function observed in the ACFDR subjects could be a function of overall warmer temperatures in Australia. To assess whether this association was independent of *CFTR* genotype, we examined white patients in the largest sample of CF subjects (CFF) who had identical *CFTR* genotypes (*F508del* homozygotes) and found that warmer temperatures remained associated with lower lung function with a similar co-efficient (−0.35; n = 6367; *p*<0.001) to that from the entire CFF sample (−0.31).

### Seasonal Difference in Temperature is also associated with Lung Function

To examine the possibility of seasonal effects, lung function data in both January and July was examined in both U.S. samples of subjects (Table 4). In the larger CFF sample, lung function was higher in January than in July (n = 2145; *p*<0.001) and there was a trend towards higher lung function in January in all four quartiles. Thus, there is a suggestion that individual patients have higher lung function in January than in July, which may reflect temperature differences between the two seasons. Of note, lung function was higher in colder temperature quartiles in both CFTSS and CFF subjects, regardless of whether sampled in January or July, suggesting the predominance of average annual temperatures over seasonal fluctuations.

**Table 4:** Comparison of Lung Function (CF-specific FEV_1_) by Temperature Quartile and Seasonal Extremes.

	Variable		Temperature Quartiles based on Entire CFTSS Population (n = 1557)				
Study Sample	Mean Annual Temperature (°F)	All Quartiles	< 49.2	49.2 – 52.0	52.1 – 58.1	>58.1	FEV_1_ ANOVA*p* value
CFTSS	Quartile n	1043	269	248	268	258	
	January Mean CF-specific FEV_1_[Temperature (°F)]	59.2±28.1[31.3±11.4]	62.7±26.9[19.6±5.4]	57.8±29.6[27.2±3.9]	60.1±27.5[31.6±4.1]	55.8±28.3[47.2±7.1]	0.033
	July Mean CF-specific FEV_1_[Temperature (°F)]	58.9±28.0[74.6±4.6]	62.4±27.0[70.4±2.2]	57.8±28.8[72.6±2.7]	60.0±27.9[76.1±2.7]	55.2±27.9[79.4±4.2]	0.023
	FEV_1_ *T* test *p* value	0.64	0.76	0.99	0.94	0.57	

	Mean Annual Temperature (°F)	All Quartiles	< 49.2	49.2 – 52.0	52.1 – 58.1	>58.1	FEV_1_ ANOVA*p* value
CFF	Quartile n	2145	467	414	524	740	
	January Mean CF-specific FEV_1_[Temperature (°F)]	54.4±27.7[33.5±12.1]	57.3±27.5[19.7±4.9]	58.2±27.3[26.1±2.6]	54.2±28.2[32.3±4.4]	50.6±27.2[47.2±7.2]	<0.001
	July Mean CF-specific FEV_1_[Temperature (°F)]	53.2±28.0[75.7±4.8]	56.3±27.7[70.7±1.8]	56.0±27.8[73.5±1.4]	53.4±28.3[75.8±3.0]	49.5±27.8[80.1±4.2]	<0.001
	FEV_1_ *T* test *p* value	<0.001	0.17	0.005	0.22	0.06	

### Temperature may act on Lung Function and *P. aeruginosa* through Independent Mechanisms

Having observed temperature to be associated with lung function and *P. aeruginosa,* we sought to determine whether temperature acts through the same or different mechanisms on these outcomes. One pathway might be that temperature affects infection rates, which in turn alters lung function. To test this, we included the presence of *P. aeruginosa* as a predictor of lung function in a mediation analysis, and found that the association between temperature and lung function decreased (Table S6: CFTSS: −0.34 to −0.29 per °F; CFF: −0.31 to −0.29; ACFDR: −0.23 to −0.18). Using these regression co-efficients, infection with *P. aeruginosa* is estimated to account for 15% of the association between temperature and lung function among CFTSS subjects (e.g., (−0.34 – −0.29)/ −0.34), 6% among CFF subjects and 22% among ACFDR subjects. The alternate pathway is that temperature affects lung function, which in turn alters the acquisition of infection. For this pathway, lung function is estimated to account for 7% of the association between temperature and *P. aeruginosa* among CFTSS subjects (e.g., (ln(1.057456)-ln(1.053488))/ln(1.057456)), 20% among CFF subjects, and 6% among ACFDR subjects (Table S7). Thus, there is limited overlap between the effects of temperature on the two clinical outcomes, suggesting that mechanisms through which temperature may act on lung function vs. on *P. aeruginosa* infection are largely different.

## Discussion

Ambient temperature has been demonstrated to affect the prevalence of both infectious and non-infectious diseases [28]. Our analysis demonstrates that CF patients living in areas with warmer annual temperatures have a higher prevalence of and an earlier age of acquisition of *P. aeruginosa*. Given a constant temperature (37°C) within the airways, the association of *P. aeruginosa* and temperature is likely mediated outside the host. Our findings may be due to increased prevalence of *P. aeruginosa* in the environment secondary to more favorable conditions for the organism in warmer climates. Regional *P. aeruginosa* biodiversity may also play a role as genotypes of soil isolates of *P. aeruginosa* differ by geographic distance [29]. Genetic differences that alter the adhesion or biofilm properties of *P. aeruginosa* may interact with local environmental features to affect the likelihood of acquisition by a CF patient [30]–[32]. Indeed, higher ambient temperatures (30°C vs. 15°C) have been shown to alter the capacity of *P. aeruginosa* to adhere to surfaces [33]. It is possible that regional differences in neonatal screening, and *P. aeruginosa* detection and prevention may lead to differences in the longitudinal development of infection [27], [34].

Regarding lung function, we estimate that patients residing in the warmest regions in the U.S. would have CF-specific lung function 10 percentile points lower than if they had resided in the coldest regions of the U.S. where mean temperatures are ∼30 degrees (°F) lower. Translated to more clinically familiar measures (NHANES FEV_1_ percentages) [35], a hypothetical 18 year old white male with CF (Height: 175cm) with an FEV_1_ of 73.5% percent living in a cold climate would be expected to have an FEV_1_ of 66.1% had he resided in a 30 degree (°F) warmer climate. Furthermore, lung function is likely associated with season, as patients tend to have higher lung function in the colder month of January compared to July. Although temperature is associated with lung function, based on the univariate regression r-value (r = 0.0069), temperature accounts for only 0.7% of the variation seen in CF lung function. Also, the mechanisms through which temperature acts on lung function are unclear, but may include infectious agents, aeroallergens, air pollution, and socio-economic status.

Our study supports the concept that infectious agents may mediate the association between temperature and lung function, at least through *P. aeruginosa*, which accounts for 6–22% of the association between temperature and lung function. Further investigation into viruses and other CF pathogens may reveal other mediators. Temperature is a predictor of pollen loads as well as mold production [36], although differences in lung function have not been seen with allergy sensitive in CF (n = 55) [37]. Higher temperatures may exacerbate the effects of air pollution [38], [39], and air pollution has already been demonstrated to be associated with lower CF lung function [11]; thus, temperature and air pollution may interact to worsen CF lung function. Geographic variation in temperature does not appear to serve as a proxy for socio-economic status in our study as the association between lung function and temperature remained robust after adjusting for significant socio-economic factors (Table S1).

Study limitations include that subjects may reside in multiple locations over a lifetime. Lung function from the most recent year of data and each subject's last known postal or zip code were used to minimize this uncertainty. Furthermore, for the U.S. subjects, 79.5% (CFTSS) and 77.6% (CFF) live within the state in which they were born with another 8% (CFTSS) and 7.3% (CFF) living within an adjacent state. A limitation of cross-sectional data is the uncertainty in measuring infection status, and hence accurate ascertainment of infection prevalence. To address this issue, longitudinal respiratory culture data were obtained in the CFTSS and ACFDR samples; it should also be noted that this longitudinal assessment may lead to temporal effects as cultures and lung function data may have been obtained for different subjects as much as 10 years apart. Another limitation is our largely retrospective culture ascertainment, thus we sought out a prospectively ascertained sample (ACFBAL). There are other confounding factors that we were unable to assess, such as co-morbidities, family support, physical activity, etc. Finally, ACFDR lung function results are subject to the caveat of applying a U.S-based FEV_1_ phenotype to an Australian population.

Our findings suggest that accounting for temperature should be considered in the design of both epidemiological studies of infection and/or lung function and clinical trials that encompass broad geographic areas. As the effects of temperature upon lung function and *P. aeruginosa* are not within patients' and clinical providers' control, geography may need to be considered when comparing the performance of CF Care Centers.

## Supporting Information

Figure S1 **Derivation of Cystic Fibrosis Twin-Sibling Study (CFTSS) sample and Cystic Fibrosis Foundation Patient Registry (CFF) sample outlining exclusions.**
(DOC)

Figure S2 **Derivation of Australian Cystic Fibrosis Data Registry (ACFDR) sample and Australian Cystic Fibrosis BAL Study (ACFBAL) sample outlining exclusions.**
(DOC)

Figure S3 **Surivial analysis for first positive respiratory culture for** ***Pseudomonas aeruginosa*** **in the CFTSS and ACFBAL samples by temperature quartile.** In both study samples, the warmer temperatures were associated with earlier acquisition of *P. aeruginosa.*
(DOC)

Table S1 **Complete Regression Analyses for Predictors of Lung Function (CF-specific FEV_1_).**
(DOC)

Table S2 **Complete Logistic Regression Analyses for Predictors of** ***P. aeruginosa*** **Infection.**
(DOC)

Table S3 **Complete Logistic Regression Analyses for Predictors of Mucoid** ***P. aeruginosa*** **Infection.**
(DOC)

Table S4 **Complete Logistic Regression Analyses for Predictors of** ***B. cepacia*** **Complex Infection.**
(DOC)

Table S5 **Study Outcomes by Temperature Quartile.**
(DOC)

Table S6 **Regression Analyses for Lung Function: Assessing the Mediation Effect of** ***P. aeruginosa.***
(DOC)

Table S7 **Regression Analyses for** ***P. aeruginosa*** **: Assessing the Mediation Effect of Lung Function.**
(DOC)

## References

[1] Kerem E, Corey M, Kerem B-S, Rommens J, Markiewicz D (1990). The relation between genotype and phenotype in cystic fibrosis--analysis of the most common mutation (deltaF508).. N Engl J Med.

[2] The Cystic Fibrosis Genotype-Phenotype Consortium (1993). Correlation between genotype and phenotype in patients with cystic fibrosis.. N Engl J Med.

[3] Koch C, Cuppens H, Rainisio M, Madessani U, Harms H (2001). European Epidemiologic Registry of Cystic Fibrosis (ERCF): comparison of major disease manifestations between patients with different classes of mutations.. Pediatr Pulmonol.

[4] Collaco JM, Blackman SM, McGready J, Naughton KM, Cutting GR (2010). Quantification of the relative contribution of environmental and genetic factors to variation in cystic fibrosis lung function.. J Pediatr.

[5] Stanke F, Becker T, Kumar V, Hedtfeld S, Becker C (2011). Genes that determine immunology and inflammation modify the basic defect of impaired ion conductance in cystic fibrosis epithelia.. J Med Genet.

[6] Collaco JM, Vanscoy L, Bremer L, McDougal K, Blackman SM (2008). Interactions between secondhand smoke and genes that affect cystic fibrosis lung disease.. JAMA.

[7] Beydon N, Amsallem F, Bellet M, Boule M, Chaussain M (2002). Pulmonary function tests in preschool children with cystic fibrosis.. Am J Respir Crit Care Med.

[8] Campbell PW, Parker RA, Roberts BT, Krishnamani MR, Phillips JA, III (1992). Association of poor clinical status and heavy exposure to tobacco smoke in patients with cystic fibrosis who are homozygous for the F508 deletion.. J Pediatr.

[9] Rubin BK (1990). Exposure of children with cystic fibrosis to environmental tobacco smoke.. N Engl J Med.

[10] Smyth A, O'Hea U, Williams G, Smyth R, Heaf D (1994). Passive smoking and impaired lung function in cystic fibrosis.. Arch Dis Child.

[11] Goss CH, Newsom SA, Schildcrout JS, Sheppard L, Kaufman JD (2004). Effect of ambient air pollution on pulmonary exacerbations and lung function in cystic fibrosis.. Am J Respir Crit Care Med.

[12] O'Connor GT, Quinton HB, Kneeland T, Kahn R, Lever T (2003). Median household income and mortality rate in cystic fibrosis.. Pediatrics.

[13] Schechter MS, McColley SA, Silva S, Haselkorn T, Konstan MW (2009). Association of socioeconomic status with the use of chronic therapies and healthcare utilization in children with cystic fibrosis.. J Pediatr.

[14] Stephenson A, Hux J, Tullis E, Austin PC, Corey M (2011). Socioeconomic status and risk of hospitalization among individuals with cystic fibrosis in Ontario, Canada.. Pediatr Pulmonol.

[15] Schechter MS, Shelton BJ, Margolis PA, Fitzsimmons SC (2001). The association of socioeconomic status with outcomes in cystic fibrosis patients in the United States.. Am J Respir Crit Care Med.

[16] Schechter MS, Margolis PA (1998). Relationship between socioeconomic status and disease severity in cystic fibrosis.. J Pediatr.

[17] Curtis JR, Burke W, Kassner AW, Aitken ML (1997). Absence of health insurance is associated with decreased life expectancy in patients with cystic fibrosis.. Am J Respir Crit Care Med.

[18] Vanscoy LL, Blackman SM, Collaco JM, Bowers A, Lai T (2007). Heritability of lung disease severity in cystic fibrosis.. Am J Respir Crit Care Med.

[19] Kulich M, Rosenfeld M, Campbell J, Kronmal R, Gibson RL (2005). Disease-specific reference equations for lung function in patients with cystic fibrosis.. Am J Respir Crit Care Med.

[20] Green DM, McDougal KE, Blackman SM, Sosnay PR, Henderson LB (2010). Mutations that permit residual CFTR function delay acquisition of multiple respiratory pathogens in CF patients.. Respir Res.

[21] Baron RM, Kenny DA (1986). The moderator-mediator variable distinction in social psychological research: conceptual, strategic, and statistical considerations.. J Pers Soc Psychol.

[22] Kenny DA (2011). Mediation.. http://davidakenny.net/cm/mediate.htm.

[23] Lipuma JJ (2005). Update on the Burkholderia cepacia complex.. Curr Opin Pulm Med.

[24] Lipuma JJ (2010). The changing microbial epidemiology in cystic fibrosis.. Clin Microbiol Rev.

[25] McKone EF, Emerson SS, Edwards KL, Aitken ML (2003). Effect of genotype on phenotype and mortality in cystic fibrosis: a retrospective cohort study.. Lancet.

[26] Kosorok MR, Jalaluddin M, Farrell PM, Shen G, Colby CE (1998). Comprehensive analysis of risk factors for acquisition of Pseudomonas aeruginosa in young children with cystic fibrosis.. Pediatr Pulmonol.

[27] Rosenfeld M, Emerson J, McNamara S, Joubran K, Retsch-Bogart G (2010). Baseline characteristics and factors associated with nutritional and pulmonary status at enrollment in the cystic fibrosis EPIC observational cohort.. Pediatr Pulmonol.

[28] Patz JA, Campbell-Lendrum D, Holloway T, Foley JA (2005). Impact of regional climate change on human health.. Nature.

[29] Cho JC, Tiedje JM (2000). Biogeography and degree of endemicity of fluorescent Pseudomonas strains in soil.. Appl Environ Microbiol.

[30] Musken M, Di FS, Dotsch A, Fischer R, Haussler S (2010). Genetic determinants of Pseudomonas aeruginosa biofilm establishment.. Microbiology.

[31] Conibear TC, Collins SL, Webb JS (2009). Role of mutation in Pseudomonas aeruginosa biofilm development.. PLoS One.

[32] Fonseca AP, Correia P, Sousa JC, Tenreiro R (2007). Association patterns of Pseudomonas aeruginosa clinical isolates as revealed by virulence traits, antibiotic resistance, serotype and genotype.. FEMS Immunol Med Microbiol.

[33] Cappello S, Guglielmino SP (2006). Effects of growth temperature on polystyrene adhesion of Pseudomonas aeruginosa ATCC 27853.. Brazilian Journal of Microbiology.

[34] Li Z, Kosorok MR, Farrell PM, Laxova A, West SE (2005). Longitudinal development of mucoid Pseudomonas aeruginosa infection and lung disease progression in children with cystic fibrosis.. JAMA.

[35] Hankinson JL, Odencrantz JR, Fedan KB (1999). Spirometric reference values from a sample of the general U.S. population.. Am J Respir Crit Care Med.

[36] U.S.Environmental Protection Agency (2008). A review of the impacts of climate variability and change on aeroallergens and their associated effects.. US EPA/600/R-06/.

[37] Hallstrand TS, Calenoff E, Becker JW, Henderson WR, Aitken ML (2004). The role of allergy in manifestations of respiratory disease in adult cystic fibrosis.. Ann Allergy Asthma Immunol.

[38] Roberts S (2004). Interactions between particulate air pollution and temperature in air pollution mortality time series studies.. Environ Res.

[39] Bloomer BJ, Stehr JW, Piety CA, Salawitch RJ, Dickerson RR (2009). Observed relationships of ozone air pollution with temperature and emissions.. Geophysical Research Letters.

