# Supplementary material for: Effect of Temperature on Cystic Fibrosis Lung Disease and Infections: A Replicated Cohort Study
Source: PLoS One. 2011 Nov 18;6(11):e27784. doi: 10.1371/journal.pone.0027784 (PMC3220679; doi:10.1371/journal.pone.0027784)
Supplement: Figure S1 — Derivation of Cystic Fibrosis Twin-Sibling Study (CFTSS) sample and Cystic Fibrosis Foundation Patient Registry (CFF) sample outlining exclusions. (DOC) [file pone.0027784.s001.doc]

**Figure S1:** CFTSS and CFF Study Sample Derivations

**CF Twin and Sibling Study**

(n = 1658)

Subjects enrolled in the CFTSS were excluded from the CFF Sample (n = 1435)

**CFF Data Registry**

(n = 24799)

(n = 23364)

Less than 6 Years of Age

(n = 132) (n = 3714)

No Lung Function Data

(n = 59) (n = 1627)

(n = 1526)

(n = 1467)

(n = 19650)

No U.S. Zip Code

(n = 43) (n = 294)

No Respiratory Culture Data

(n = 0) (n = 993)

**CFTSS**

**Sample**

(n = 1378)

(n = 18023)

(n = 17030)

**CFF**

**Sample**

(n = 16439)

(n = 1467)

(n = 1424)

Active Smokers

(n = 46) (n = 297)

(n = 16736)
