# Supplementary material for: Effect of Temperature on Cystic Fibrosis Lung Disease and Infections: A Replicated Cohort Study
Source: PLoS One. 2011 Nov 18;6(11):e27784. doi: 10.1371/journal.pone.0027784 (PMC3220679; doi:10.1371/journal.pone.0027784)
Supplement: Figure S2 — Derivation of Australian Cystic Fibrosis Data Registry (ACFDR) sample and Australian Cystic Fibrosis BAL Study (ACFBAL) sample outlining exclusions. (DOC) [file pone.0027784.s002.doc]

**Figure S2:** ACFDR and ACFBAL Study Sample Derivations

**Australian CF Data Registry**

(n =3789)

**Australian CF BAL Study**

(n = 168)

Less than 6 Years of Age

(n =595)

No Lung Function Data

(n =1280)

(n = 3194)

(n = 1914)

No Respiratory Culture Data

(n = 99) (n = 0)

**ACFDR**

**Sample**

(n = 1801)

**ACFBAL**

**Sample**

(n = 167)

(n = 1815)

No Postal Code

(n = 14) (n = 1)

(n = 168)
