# Supplementary figures and images for: Effect of Temperature on Cystic Fibrosis Lung Disease and Infections: A Replicated Cohort Study
Source: PLoS One. 2011 Nov 18;6(11):e27784. doi: 10.1371/journal.pone.0027784 (PMC3220679; doi:10.1371/journal.pone.0027784)

**Figure S3.** Kaplan-Meier curves for remaining free of *P. aeruginosa* on respiratory cultures


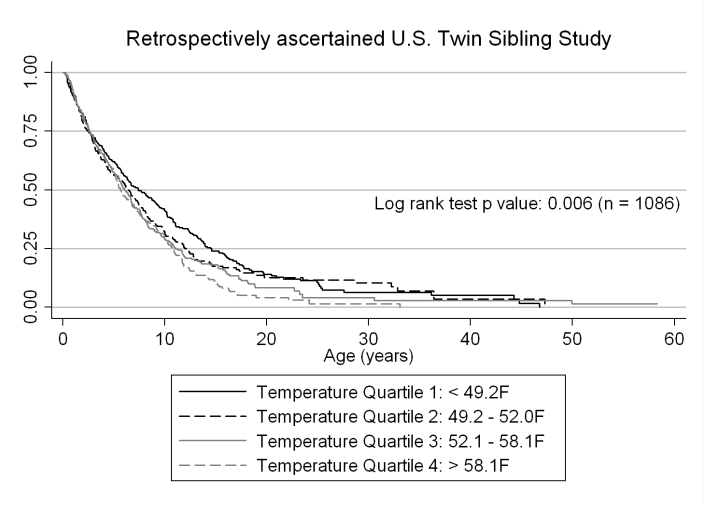

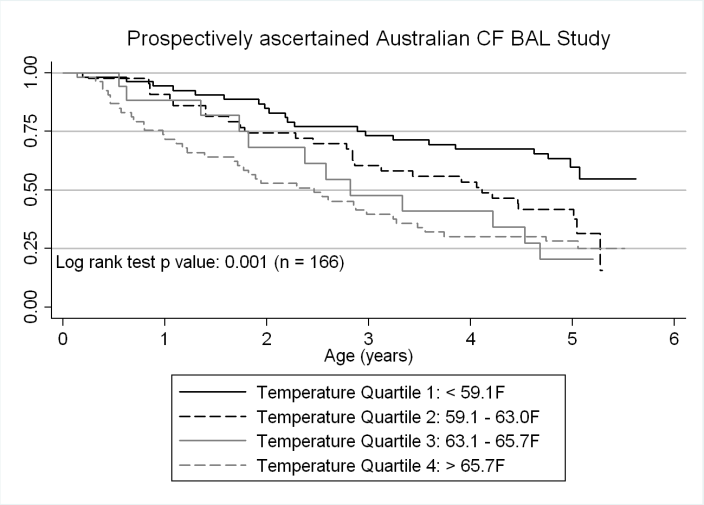

Supplement: Figure S3 — Surivial analysis for first positive respiratory culture for Pseudomonas aeruginosa in the CFTSS and ACFBAL samples by temperature quartile. In both study samples, the warmer temperatures were associated with earlier acquisition of P. aeruginosa. (DOC) [file pone.0027784.s003.doc]
