# Supplementary material for: Effect of Temperature on Cystic Fibrosis Lung Disease and Infections: A Replicated Cohort Study
Source: PLoS One. 2011 Nov 18;6(11):e27784. doi: 10.1371/journal.pone.0027784 (PMC3220679; doi:10.1371/journal.pone.0027784)
Supplement: Table S1 — Complete Regression Analyses for Predictors of Lung Function (CF-specific FEV1). (DOC) [file pone.0027784.s004.doc]

**Table S1.** Complete Regression Analyses for Predictors of Lung Function (CF-specific FEV_1_)

|  | **Variable** | **CFTSS** | | | **CFF** | | **ACFDR** |
| --- | --- | --- | --- | --- | --- | --- | --- |
|  | **Co-efficient**  **[95%CI]**  **(*p* value, n if applicable)** | **Univariate Regression** | **Preliminary Multivariate Model** | **Final Multivariate Model** | **Replication of Final Model** | **Replication of Final Model (White, *F508del* homozygotes)** | **Replication of Final Model** |
|  | Multivariate Sample n | - | 1167 | 1313 | 15174 | 6367 | 1791 |
|  | Multivariate Model *p* Value | - | <0.001 | <0.001 | <0.001 | <0.001 | 0.09 |
|  | Multivariate Model r | - | 0.22 | 0.19 | 0.21 | 0.20 | 0.05 |
| Demographics | Sex  (0=male, 1=female) | 1.98  [-1.01, 4.98]  (0.19, n = 1378) |  |  |  |  |  |
|  | CFTR Genotype  (# *F508del* mutations) | 1.43  [-1.49, 4.36]  (0.33, n = 1371) |  |  |  |  |  |
|  | Race/Ethnicity  (0=White, 1=Non-white) | -5.50  [-12.62, 1.63]  (0.13, n = 1378) |  |  |  |  |  |
|  | Age at time of lung function test  (yrs) | -0.36  [-0.54, -0.18]  (<0.001, n = 1378) | -0.31  [-0.52, -0.09]  (0.005) | -0.40  [-0.58, -0.22]  (<0.001) | -0.27  [-0.32, -0.21]  (<0.001) | -0.27  [-0.35, -0.19]  (<0.001) | -0.05  [-0.16, 0.05]  (0.32) |
|  | Age at Diagnosis  (yrs) | 0.19  [-0.10, 0.48]  (0.20, n = 1378) |  |  |  |  |  |
| Household Factors | Secondhand Smoke  (0=Not exposed, 1=exposed) | -3.25  [-6.70, 0.22]  (0.07, n = 1313) |  |  |  |  |  |
|  | Maternal Education  (Scale: 1-4) | 2.61  [0.82, 4.40]  (0.004, n = 1296) | 1.25  [-0.61, 3.11]  (0.19) |  |  |  |  |
|  | Log Income  (log $) | 10.42  [0.21, 20.63]  (0.046, n = 1378) | 1.35  [-10.11, 12.82]  (0.82) |  |  |  |  |
|  | Insurance Status  (0=Any Insurance, 1=No Insurance) | -9.63  [-20.57, 1.30]  (0.08, n = 1357) |  |  |  |  |  |
|  | Insurance Status  (0=Private, 1=Public) | -5.34  [-8.60, -2.08]  (0.001, n = 1319) | -6.44  [-10.06, -2.82]  (0.001) | -6.43  [-9.68, -3.19]  (<0.001) | -9.11  [-10.44, -7.79]  (<0.001) | -8.26  [-9.90, -6.62]  (<0.001) | Not Available |
|  | Household Density (persons/household) | 1.50  [0.46, 2.54]  (0.005, n = 1275) | 0.97  [-0.19, 2.14]  (0.10) |  |  |  |  |
| Geographic Factors  (by residential zip code) | PM_2.5_ level  (μg/m^3^) | -0.63  [-1.64, 0.38]  (0.22, n = 677) |  |  |  |  |  |
|  | Log Elevation  (log m) | 3.29  [0.47, 6.10]  (0.022, n = 1372) | 1.57  [-1.68, 4.82]  (0.34) |  |  |  |  |
|  | Relative Humidity  (%) | -0.02  [-0.32, 0.28]  (0.91, n = 1372) |  |  |  |  |  |
|  | Temperature  (°F) | -0.30  [-0.53, -0.06]  (0.015, n = 1372) | -0.31  [-0.58, -0.04]  (0.023) | -0.34  [-0.57, -0.10]  (0.005) | -0.31  [-0.41, -0.21]  (<0.001) | -0.35  [-0.46, -0.23]  (<0.001) | -0.23  [-0.47, 0.01]  (0.057) |
|  | Log Distance from Care  (log Km) | -1.23  [-4.23, 1.76]  (0.42, n = 1377) |  |  |  |  |  |
|  | Log Population Density  (log persons/km^2^) | -0.30  [-2.44, 1.85]  (0.79, n = 1364) |  |  |  |  |  |
