# Supplementary material for: Effect of Temperature on Cystic Fibrosis Lung Disease and Infections: A Replicated Cohort Study
Source: PLoS One. 2011 Nov 18;6(11):e27784. doi: 10.1371/journal.pone.0027784 (PMC3220679; doi:10.1371/journal.pone.0027784)
Supplement: Table S2 — Complete Logistic Regression Analyses for Predictors of P. aeruginosa Infection. (DOC) [file pone.0027784.s005.doc]

**Table S2. Complete Logistic Regression Analyses for Predictors of *P. aeruginosa* Infection**

|  | **Variable** | **CFTSS** | | | **CFF** | **ACFDR** | **ACFBAL** |
| --- | --- | --- | --- | --- | --- | --- | --- |
|  | **Odds Ratio**  **[95%CI]**  **(*p* value, n if applicable)** | **Univariate Regression** | **Preliminary Multivariate Model** | **Final Multivariate Model** | **Replication of Final Model** | **Replication of Final Model** | **Replication of Final Model** |
|  | Multivariate Sample n | - | 627 | 1366 | 13956 | 1474 | 166 |
|  | Multivariate Model *p* Value | - | <0.001 | <0.001 | <0.001 | <0.001 | 0.036 |
|  | Multivariate Model r | - | 0.44 | 0.42 | 0.31 | 0.41 | 0.21 |
| Demographics | Sex  (0=male, 1=female) | 0.93  [0.67, 1.28]  (0.66, n = 1378) |  |  |  |  |  |
|  | CFTR Genotype  (# *F508del* mutations) | 2.13  [1.64, 2.77]  (<0.001, n = 1371) | 2.34  [1.53, 3.58]  (<0.001) | 2.05  [1.55, 2.72]  (<0.001) | 1.23  [1.16, 1.31]  (<0.001) | 1.45  [1.16, 1.81]  (0.001) | 1.13  [0.60, 2.14]  (0.71) |
|  | Race/Ethnicity  (0=White, 1=Non-white) | 0.80  [0.42, 1.53]  (0.50, n = 1378) |  |  |  |  |  |
|  | Age at time of last respiratory culture  (yrs) | 1.09  [1.05, 1.13]  (<0.001, n = 1378) | 1.20  [1.12, 1.27]  (<0.001) | 1.19  [1.14, 1.25]  (<0.001) | 1.09  [1.08, 1.10]  (<0.001) | 1.16  [1.14, 1.19]  (<0.001) | 1.07  [0.73, 1.56]  (0.74) |
|  | Age at Diagnosis  (yrs) | 0.96  [0.94, 0.98]  (<0.001, n = 1378) | 0.86  [0.80, 0.92]  (<0.001) | 0.84  [0.80, 0.88]  (<0.001) | 0.94  [0.93, 0.94]  (<0.001) | 0.91  [0.88, 0.94]  (<0.001) | 1.18  [0.00, 407.4]  (0.96) |
| Household Factors | Secondhand Smoke  (0=Not exposed, 1=exposed) | 1.20  [0.79, 1.82]  (0.40, n = 1313) |  |  |  |  |  |
|  | Maternal Education  (Scale: 1-4) | 0.88  [0.74, 1.06]  (0.19, n = 1296) |  |  |  |  |  |
|  | Log Income  (log $) | 1.19  [0.39, 3.60]  (0.76, n = 1378) |  |  |  |  |  |
|  | Insurance Status  (0=Any Insurance, 1=No Insurance) | 0.51  [0.22, 1.18]  (0.12, n = 1357) |  |  |  |  |  |
|  | Insurance Status  (0=Private, 1=Public) | 1.14  [0.77, 1.69]  (0.51, n = 1319) |  |  |  |  |  |
|  | Household Density (persons/household) | 0.85  [0.77, 0.94]  (0.001, n = 1275) | 0.99  [0.81, 1.21]  (0.91) |  |  |  |  |
| Geographic Factors  (by residential zip code) | PM_2.5_ level  (μg/m^3^) | 1.12  [1.01, 1.23]  (0.033, n = 677) | 1.10  [0.99, 1.23]  (0.07) |  |  |  |  |
|  | Log Elevation  (log m) | 1.04  [0.77, 1.42]  (0.79, n = 1372) |  |  |  |  |  |
|  | Relative Humidity  (%) | 0.98  [0.94, 1.01]  (0.17, n = 1372) |  |  |  |  |  |
|  | Temperature  (°F) | 1.05  [1.02, 1.08]  (0.002, n = 1372) | 1.06  [1.02, 1.10]  (0.005) | 1.06  [1.03, 1.09]  (<0.001) | 1.02  [1.01, 1.02]  (<0.001) | 1.05  [1.02, 1.08]  (0.002) | 1.09  [1.03, 1.16]  (0.003) |
|  | Log Distance from Care  (log Km) | 1.41  [1.06, 1.88]  (0.019, n = 1377) | 1.36  [0.86, 2.15]  (0.19) |  |  |  |  |
|  | Log Population Density  (log persons/km^2^) | 0.86  [0.68, 1.08]  (0.19, n = 1364) |  |  |  |  |  |
