# Supplementary material for: Effect of Temperature on Cystic Fibrosis Lung Disease and Infections: A Replicated Cohort Study
Source: PLoS One. 2011 Nov 18;6(11):e27784. doi: 10.1371/journal.pone.0027784 (PMC3220679; doi:10.1371/journal.pone.0027784)
Supplement: Table S3 — Complete Logistic Regression Analyses for Predictors of Mucoid P. aeruginosa Infection. (DOC) [file pone.0027784.s006.doc]

**Table S3.** Complete Logistic Regression Analyses for Predictors of Mucoid *P. aeruginosa* Infection

|  | **Variable** | **CFTSS** | | | **CFF** |
| --- | --- | --- | --- | --- | --- |
|  | **Odds Ratio**  **[95%CI]**  **(*p* value, n if applicable)** | **Univariate Regression** | **Preliminary Multivariate Model** | **Final Multivariate Model** | **Replication of Final Model** |
|  | Multivariate Sample n | - | 1271 | 1378 | 16121 |
|  | Multivariate Model *p* Value | - | <0.001 | <0.001 | <0.001 |
|  | Multivariate Model r | - | 0.34 | 0.34 | 0.28 |
| Demographics | Sex  (0=male, 1=female) | 1.04  [0.83, 1.30]  (0.75, n = 1378) |  |  |  |
|  | CFTR Genotype  (# *F508del* mutations) | 1.17  [1.96, 1.42]  (0.13, n = 1371) |  |  |  |
|  | Race/Ethnicity  (0=White, 1=Non-white) | 0.71  [0.44, 1.13]  (0.15, n = 1378) |  |  |  |
|  | Age at time of last respiratory culture  (yrs) | 1.13  [1.09, 1.17]  (<0.001, n = 1378) | 1.12  [1.08, 1.16]  (<0.001) | 1.13  [1.09, 1.17]  (<0.001) | 1.07  [1.06, 1.07]  (<0.001) |
|  | Age at Diagnosis  (yrs) | 0.99  [0.98, 1.01]  (0.54, n = 1378) |  |  |  |
| Household Factors | Secondhand Smoke  (0=Not exposed, 1=exposed) | 1.22  [0.92, 1.62]  (0.17, n = 1313) |  |  |  |
|  | Maternal Education  (Scale: 1-4) | 0.93  [0.83, 1.05]  (0.25, n = 1296) |  |  |  |
|  | Log Income  (log $) | 1.44  [0.63, 3.25]  (0.39, n = 1378) |  |  |  |
|  | Insurance Status  (0=Any Insurance, 1=No Insurance) | 1.58  [0.72, 3.45]  (0.25, n = 1357) |  |  |  |
|  | Insurance Status  (0=Private, 1=Public) | 1.01  [0.78, 1.32]  (0.93, n = 1319) |  |  |  |
|  | Household Density (persons/household) | 0.77  [0.69, 0.85]  (<0.001, n = 1275) | 0.94  [0.85, 1.04]  (0.22) |  |  |
| Geographic Factors  (by residential zip code) | PM_2.5_ level  (μg/m^3^) | 1.07  [0.99, 1.15]  (0.07, n = 677) |  |  |  |
|  | Log Elevation  (log m) | 0.80  [0.64, 0.99]  (0.040, n = 1372) | 0.86  [0.68, 1.08]  (0.20) |  |  |
|  | Relative Humidity  (%) | 0.99  [0.96, 1.01]  (0.38, n = 1372) |  |  |  |
|  | Temperature  (°F) | 1.01  [0.99, 1.03]  (0.17, n = 1372) |  |  |  |
|  | Log Distance from Care  (log Km) | 1.20  [0.97, 1.50]  (0.10, n = 1377) |  |  |  |
|  | Log Population Density  (log persons/km^2^) | 1.09  [0.93, 1.29]  (0.29, n = 1364) |  |  |  |
