# Supplementary material for: Effect of Temperature on Cystic Fibrosis Lung Disease and Infections: A Replicated Cohort Study
Source: PLoS One. 2011 Nov 18;6(11):e27784. doi: 10.1371/journal.pone.0027784 (PMC3220679; doi:10.1371/journal.pone.0027784)
Supplement: Table S4 — Complete Logistic Regression Analyses for Predictors of B. cepacia Complex Infection. (DOC) [file pone.0027784.s007.doc]

**Table S4.** Complete Logistic Regression Analyses for Predictors of *B. cepacia* Complex Infection

|  | **Variable** | **CFTSS** | | | **CFF** | **ACFDR** |
| --- | --- | --- | --- | --- | --- | --- |
|  | **Odds Ratio**  **[95%CI]**  **(*p* value, n if applicable)** | **Univariate Regression** | **Preliminary Multivariate Model** | **Final Multivariate Model** | **Replication of Final Model** | **Replication of Final Model** |
|  | Multivariate Sample n | - | 1275 | 1275 | 11613 | 1801 |
|  | Multivariate Model *p* Value | - | <0.001 | <0.001 | <0.001 | 0.07 |
|  | Multivariate Model r | - | 0.20 | 0.20 | 0.07 | 0.06 |
| Demographics | Sex  (0=male, 1=female) | 0.86  [0.56, 1.32]  (0.49, n = 1378) |  |  |  |  |
|  | CFTR Genotype  (# *F508del* mutations) | 1.22  [0.88, 1.70]  (0.24, n = 1371) |  |  |  |  |
|  | Race/Ethnicity  (0=White, 1=Non-white) | 1.31  [0.59, 2.92]  (0.51, n = 1378) |  |  |  |  |
|  | Age at time of last respiratory culture  (yrs) | 1.05  [1.03, 1.07]  (<0.001, n = 1378) | 1.03  [1.01, 1.05]  (0.012) | 1.03  [1.01, 1.05]  (0.012) | 1.01  [1.01, 1.02]  (0.001) | 1.02  [1.00, 1.03]  (0.07) |
|  | Age at Diagnosis  (yrs) | 0.99  [0.94, 1.04]  (0.59, n = 1378) |  |  |  |  |
| Household Factors | Secondhand Smoke  (0=Not exposed, 1=exposed) | 0.81  [0.50, 1.33]  (0.41, n = 1313) |  |  |  |  |
|  | Maternal Education  (Scale: 1-4) | 1.05  [0.84, 1.31]  (0.69, n = 1296) |  |  |  |  |
|  | Log Income  (log $) | 0.95  [0.19, 4.70]  (0.76, n = 1378) |  |  |  |  |
|  | Insurance Status  (0=Any Insurance, 1=No Insurance) | 1.91  [0.72, 5.05]  (0.19, n = 1357) |  |  |  |  |
|  | Insurance Status  (0=Private, 1=Public) | 0.78  [0.48, 1.27]  (0.32, n = 1319) |  |  |  |  |
|  | Household Density (persons/household) | 0.73  [0.61, 0.86]  (<0.001, n = 1275) | 0.82  [0.68, 0.99]  (0.040) | 0.82  [0.68, 0.99]  (0.040) | 0.97  [0.90, 1.06]  (0.50) | Not Available |
| Geographic Factors  (by residential zip code) | PM_2.5_ level  (μg/m^3^) | 0.98  [0.87, 1.12]  (0.81, n = 677) |  |  |  |  |
|  | Log Elevation  (log m) | 0.85  [0.59, 1.23]  (0.40, n = 1372) |  |  |  |  |
|  | Relative Humidity  (%) | 1.00  [0.96, 1.05]  (0.88, n = 1372) |  |  |  |  |
|  | Temperature  (°F) | 1.00  [0.97, 1.03]  (0.88, n = 1372) |  |  |  |  |
|  | Log Distance from Care  (log Km) | 1.06  [0.71, 1.59]  (0.76, n = 1377) |  |  |  |  |
|  | Log Population Density  (log persons/km^2^) | 1.28  [0.95, 1.72]  (0.10, n = 1364) |  |  |  |  |
