# Supplementary material for: Effect of Temperature on Cystic Fibrosis Lung Disease and Infections: A Replicated Cohort Study
Source: PLoS One. 2011 Nov 18;6(11):e27784. doi: 10.1371/journal.pone.0027784 (PMC3220679; doi:10.1371/journal.pone.0027784)
Supplement: Table S5 — Study Outcomes by Temperature Quartile. (DOC) [file pone.0027784.s008.doc]

**Table S5.** Study Outcomes by Temperature Quartile

|  |  | **Temperature Quartiles (°F)^1^** | | | |  |
| --- | --- | --- | --- | --- | --- | --- |
| **Variable** | **Study Sample** | **Coldest** | **🡨 Temperate 🡪** | | **Warmest** | **Chi square or ANOVA**  ***p* value** |
| U.S. Temperature Quartiles | CFTSS/CFF | < 49.2 | 49.2 – 52.0 | 52.1 – 58.1 | > 58.1 | - |
| Australian Temperature Quartiles | ACFDR/ACFBAL | < 59.1 | 59.1 – 63.0 | 63.1 – 65.7 | > 65.7 | - |
| Quartile n | CFTSS | 373 | 319 | 344 | 336 | - |
|  | CFF | 3619 | 2892 | 3719 | 5302 | - |
|  | ACFDR | 378 | 488 | 447 | 478 | - |
|  | ACFBAL | 53 | 43 | 17 | 53 | - |
| *P. aeruginosa* (% Positive) | CFTSS | 85.3 | 85.0 | 88.4 | 92.9 | 0.005 |
|  | CFF | 57.6 | 60.8 | 59.1 | 62.8 | <0.001 |
|  | ACFDR | 72.8 | 81.2 | 82.1 | 83.3 | 0.001 |
|  | ACFBAL | 39.6 | 65.1 | 70.6 | 73.6 | 0.002 |
| Mucoid *P. aeruginosa* (% Positive) | CFTSS | 60.3 | 61.1 | 59.6 | 64.3 | 0.61 |
|  | CFF | 40.7 | 43.9 | 41.1 | 44.5 | <0.001 |
| *B. cepacia* complex (% Positive) | CFTSS | 8.0 | 8.2 | 6.7 | 6.9 | 0.83 |
|  | CFF | 3.5 | 3.8 | 3.4 | 3.0 | 0.24 |
|  | ACFDR | 7.1 | 5.5 | 6.9 | 8.0 | 0.52 |
| *P. aeruginosa* Age of Acquisition (Yrs) | CFTSS | 7.6 ± 7.5  (n = 252) | 6.2 ± 6.5  (n = 206) | 6.3 ± 5.8  (n = 244) | 6.3 ± 5.0  (n = 214) | 0.04 |
|  | ACFBAL | 2.6 ± 1.5  (n = 21) | 2.7 ± 1.5  (n = 28) | 2.6 ± 1.4  (n = 12) | 1.8 ± 1.3  (n = 39) | 0.04 |
| Mucoid *P. aeruginosa* Age of Acquisition (Yrs) | CFTSS | 11.7 ± 7.9  (n = 216) | 10.6 ± 7.8  (n = 187) | 11.7 ± 8.7  (n = 193) | 11.0 ± 7.3  (n = 198) | 0.44 |
| *B. cepacia* complex Age of Acquisition (Yrs) | CFTSS | 15.0 ± 8.6  (n = 29) | 14.0 ± 10.2  (n = 25) | 13.0 ± 7.8  (n = 23) | 13.0 ± 7.5  (n = 23) | 0.82 |
| Lung Function (CF-specific FEV_1_ Percentile) | CFTSS | 71.2 ± 25.0 | 69.5 ± 27.1 | 70.6 ± 25.2 | 66.3 ± 27.6 | 0.07 |
|  | CFF | 68.4 ± 25.7 | 67.3 ± 25.9 | 64.8 ± 26.6 | 62.6 ± 26.7 | <0.001 |
|  | ACFDR | 61.7 ± 25.2 | 65.1 ± 24.0 | 64.4 ± 24.2 | 59.8 ± 24.6 | 0.003 |

^1^Temperature quartiles for U.S. samples based on entire CFTSS population (n = 1557); temperature quartiles for Australian samples based on entire ACFDR population (n = 3635).
