# Supplementary material for: Effect of Temperature on Cystic Fibrosis Lung Disease and Infections: A Replicated Cohort Study
Source: PLoS One. 2011 Nov 18;6(11):e27784. doi: 10.1371/journal.pone.0027784 (PMC3220679; doi:10.1371/journal.pone.0027784)
Supplement: Table S6 — Regression Analyses for Lung Function: Assessing the Mediation Effect of P. aeruginosa. (DOC) [file pone.0027784.s009.doc]

**Table S6.** Regression Analyses for Lung Function: Assessing the Mediation Effect of *P. aeruginosa*

|  | **CFTSS** | | **CFF** | | **ACFDR** | |
| --- | --- | --- | --- | --- | --- | --- |
| **Co-efficient**  **[95%CI]**  **(*p* value)** | **Final Model** | **Final Model**  **With**  ***P. aeruginosa*** | **Final Model** | **Final Model**  **With**  ***P. aeruginosa*** | **Final Model** | **Final Model**  **With**  ***P. aeruginosa*** |
| Multivariate Sample n | 1313 | 1313 | 15174 | 15174 | 1791 | 1791 |
| Multivariate Model *p* Value | <0.001 | <0.001 | <0.001 | <0.001 | 0.09 | <0.001 |
| Multivariate Model r | 0.19 | 0.23 | 0.21 | 0.24 | 0.05 | 0.14 |
| Age at time of lung function test  (yrs) | -0.40  [-0.58, -0.22]  (<0.001) | -0.34  [-0.51, -0.16]  (<0.001) | -0.27  [-0.32, -0.21]  (<0.001) | -0.18  [-0.24, -0.13]  (<0.001) | -0.05  [-0.16, 0.05]  (0.32) | -0.02  [-0.09, 0.13]  (0.68) |
| Insurance Status  (0=Private, 1=Public) | -6.43  [-9.68, -3.19]  (<0.001) | -6.07  [-9.29, -2.85]  (<0.001) | -9.11  [-10.44, -7.79]  (<0.001) | -8.87  [-10.21, -7.53]  (<0.001) | Not Available | Not Available |
| Temperature  (°F) | -0.34  [-0.57, -0.10]  (0.005) | -0.29  [-0.52, -0.06]  (0.014) | -0.31  [-0.41, -0.21]  (<0.001) | -0.29  [-0.39, -0.19]  (<0.001) | -0.23  [-0.47, 0.01]  (0.06) | -0.18  [-0.41, 0.06]  (0.15) |
| *P. aeruginosa* (0=Negative, 1 = Positive) | - | -10.72  [-14.19, -7.24]  (<0.001) | - | -6.92  [-7.95, -5.88]  (<0.001) | - | -8.04  [-10.98, -5.10]  (<0.001) |
