# Supplementary material for: Effect of Temperature on Cystic Fibrosis Lung Disease and Infections: A Replicated Cohort Study
Source: PLoS One. 2011 Nov 18;6(11):e27784. doi: 10.1371/journal.pone.0027784 (PMC3220679; doi:10.1371/journal.pone.0027784)
Supplement: Table S7 — Regression Analyses for P. aeruginosa : Assessing the Mediation Effect of Lung Function. (DOC) [file pone.0027784.s010.doc]

**Table S7.** Regression Analyses for *P. aeruginosa*: Assessing the Mediation Effect of Lung Function

|  | **CFTSS** | | **CFF** | | **ACFDR** | |
| --- | --- | --- | --- | --- | --- | --- |
| **Co-efficient**  **[95%CI]**  **(*p* value)** | **Final Model** | **Final Model**  **With**  **Lung Function** | **Final Model** | **Final Model**  **With**  **Lung Function** | **Final Model** | **Final Model**  **With**  **Lung Function** |
| Multivariate Sample n | 1366 | 1366 | 13956 | 13956 | 1474 | 1474 |
| Multivariate Model *p* Value | <0.001 | <0.001 | <0.001 | <0.001 | <0.001 | <0.001 |
| Multivariate Model r | 0.42 | 0.45 | 0.31 | 0.33 | 0.41 | 0.43 |
| CFTR Genotype  (# *F508del* mutations) | 2.05  [1.55, 2.72]  (<0.001) | 2.06  [1.55, 2.75]  (<0.001) | 1.23  [1.16, 1.31]  (<0.001) | 1.24  [1.16, 1.32]  (<0.001) | 1.45  [1.16, 1.81]  (0.001) | 1.42  [1.13, 1.79]  (0.002) |
| Age at time of last respiratory culture  (yrs) | 1.19  [1.14, 1.25]  (<0.001) | 1.18  [1.13, 1.24]  (<0.001) | 1.09  [1.08, 1.10]  (<0.001) | 1.09  [1.08, 1.10]  (<0.001) | 1.16  [1.14, 1.19]  (<0.001) | 1.17  [1.14, 1.20]  (<0.001) |
| Age at Diagnosis  (yrs) | 0.84  [0.80, 0.88]  (<0.001) | 0.84  [0.80, 0.88]  (<0.001) | 0.94  [0.93, 0.94]  (<0.001) | 0.94  [0.93, 0.95]  (<0.001) | 0.91  [0.88, 0.94]  (<0.001) | 0.91  [0.88, 0.94]  (<0.001) |
| Temperature  (°F) | 1.06  [1.03, 1.09]  (<0.001) | 1.05  [1.02, 1.09]  (0.001) | 1.02  [1.01, 1.02]  (<0.001) | 1.01  [1.01, 1.02]  (0.001) | 1.05  [1.02, 1.08]  (0.002) | 1.05  [1.01, 1.08]  (0.005) |
| Lung Function (CF-specific FEV_1_ Percentile) | - | 0.98  [0.97, 0.99]  (<0.001) | - | 0.99  [0.99, 0.99]  (<0.001) | - | 0.98  [0.98, 0.99]  (<0.001) |
